# Assessment of knowledge and foot self-care practices among diabetes mellitus patients in a tertiary care centre in Makkah, Saudi Arabia: a cross-sectional analytical study

**DOI:** 10.11604/pamj.2021.40.123.30113

**Published:** 2021-10-29

**Authors:** Abrar Ali Wazqar, Meaad Mohammed Baatya, Fahad Saqib Lodhi, Adeel Ahmed Khan

**Affiliations:** 1 Ministry of Health, Makkah, Saudi Arabia,; 2 Saudi Board of Preventive Medicine, Ministry of Health, Makkah, Saudi Arabia

**Keywords:** Diabetes, foot, self-care, diabetes mellitus, knowledge, practice

## Abstract

**Introduction:**

diabetic foot is a common long-term complication of uncontrolled diabetes mellitus. Knowledge about foot self-care practices among diabetic patients in Saudi Arabia is limited. Thus, the aim of this study was to assess the level of patients´ knowledge and practices regarding self-care of diabetic foot among diabetic patients.

**Methods:**

a cross-sectional analytical study was conducted using a sample of diabetic patients attending outpatient clinics at Heraa Diabetic Center, Ministry of Health, Makkah City, Saudi Arabia from June 2020 to July 2020. The participants were interviewed through a self-administered questionnaire inquiring sociodemographic factors, patients´ knowledge and practices regarding foot self-care.

**Results:**

a total of 409 patients were included in this study. Respondents' ages ranged between 6 and 75 years with a mean of 42.5 years and standard deviation of 13.9 years. Females represented 51.1% of the participants. Type 2 diabetes represented 85.6% of the responses. Overall, most of the participants (72.4%) had a poor level of knowledge whereas only 4.2% expressed a good level of knowledge. Being employed (P = 0.046), type II diabetes patients (P = 0.047) and those whose main source of information was health staff (P = 0.026) were more knowledgeable compared to their peers. Almost two-thirds (63.3%) of patients showed a poor level of practice related to diabetic foot self-care.

**Conclusion:**

low level of knowledge and practice of foot care are prevalent among diabetic patients attending outpatient clinics at Heraa Diabetic Center. Designed interventions should be implemented to enhance diabetic foot self-care.

## Introduction

The prevalence of diabetes mellitus (DM) in Saudi Arabia dramatically increased from 3.4% in 1996 to more than 20% in recent years which is attributed to change in lifestyle. Saudi Arabia, ranked the seventh among top ten countries in regards to diabetes mellitus prevalence [1]. Diabetes-associated complications increase the burden of disease globally due to prolonged morbidity. Around 366 million people have developed diabetes in 2011 and 552 million are expected to be diabetic by 2030 [2]. People aged 40-59 years old are mostly affected and about 183 million (50%) diabetic patients are undiagnosed. It is estimated that about seven million of the Saudi population are diabetic and almost about three million are pre-diabetics [3]. The spread of sedentary lifestyle and adoption of unhealthy dietary habits high in refined carbohydrates and fat are driving an increase in the number of people with obesity-related type 2 diabetes.

Diabetic foot is one of the most common long-term complications of uncontrolled diabetes mellitus and it accounts for 80% of non-traumatic foot amputations [4]. Despite many educational campaigns, knowledge about foot self-care practices among diabetic patients in Saudi Arabia is limited [5]. The escalating diabetes prevalence combined with its long-term complications will heavily increase the burden on the healthcare system. Therefore, education on self-care for individuals with diabetes and complications prevention would reduce the burden of the disease in the future [6].

The self-care concept can be related to the practice of activities that individuals initiate and perform on their own behalf in order to maintain life, health, and well-being [7]. The patient's own role in diabetes management and recognition of the need to educate individuals about self-care are considered vital pillars in diabetes treatment [8], which stressed the importance of developing effective patient education programs to maintain the health and quality of life of individuals with diabetes. Managing the daily care of diabetes seems to be a challenging task for many patients [6,9], and a patient´s ability to be involved in the daily routine of diabetes self-care seems to be grounded in psychological, motivational as well as educational factors [10].

Multiple systematic reviews and meta-analyses showed that promoting self-care awareness through educational and behavioral strategies improves individuals´ practices and, consequently, results in better health outcomes [10,11]. Foot self-care practices such as daily inspection, hygiene practices, shoes selection, and seeking professional treatment for wound and ulcers can decrease the risk of foot complications [5]. Studies showed that awareness about proper foot care reduced complications, admission, and amputation rates [6,7]. In Saudi Arabia, 55.2% of the diabetic patients developed complications related to diabetic foot with only 41% having previous knowledge about these complications. About 40% indicated they can treat the wound by themselves, while only 36% stated they read about foot care [12]. Thus, this study aimed to assess knowledge and practices regarding foot-care and self-care practices among diabetic patients in Makkah, Saudi Arabia

## Methods

This study was a cross-sectional analytical study including a sample of diabetic patients attending outpatient clinics at Heraa Diabetic Center, Ministry of Health located in Makkah City, during the period from June 2020 to July 2020. Inclusion criteria were patients who were diagnosed with type 1 or type 2 diabetes mellitus type for at least 6 months, those who had ever developed foot ulcerations, those aged over 18 years, those with good mental status, and psychological status and those who were literate. Patients with physical disabilities, refused to sign informed consent and those who cannot communicate in Arabic or English language were excluded.

The number of participants required to estimate the level of knowledge was calculated using this equation:


$$n=\frac{P(1-P)z^{2}}{d^{2}}$$

The expected good level of patients´ knowledge was approximately 41% based on previous study in Saudi Arabia [12]. Thus, at the confidence level of 95% and estimation error of 0.05, the sample size was calculated as follows:


$$n=\frac{0.41(1-0.41)1.96^{2}}{0.05^{2}}=372\text{ participants}$$

After adding 10% to compensate for item non-response, the final sample size was 409 participants. The patients were selected from the waiting list of patients. Selection of patients was conducted randomly through a systematic random sampling technique.

A structured questionnaire in Arabic was used to collect study data. The questionnaire consisted of three sections, section A contained questions about sociodemographic factors of the patients, section B contained questions about patients´ knowledge about diabetic foot self-care, and section C included questions about patients´ practices regarding diabetic foot self-care. The knowledge score was calculated out of 11 points, <60% was categorized as low level of knowledge, 60 - 80% was categorized as medium level of knowledge, and >80% was categorized as good level of knowledge. Based on the 11 questions related to practice, a score was calculated. Patients scored <60% were categorized as poor practice, 60 - 80% were categorized as satisfactory practice, and >80% were categorized as good practice.

The questionnaire was adopted from previous similar studies [13,14] and translated into Arabic by experts (forward-backward translation). The final version of the questionnaire was validated by a panel of experts. Cronbach alpha value was 0.78. Due to the circumstances of COVID-19 pandemic, the questionnaire was distributed through an online link. The patients were called by telephone and an informed consent was gained after they received all the important information. Next, a link to the questionnaire was sent to their phones and the responses were collected automatically into excel sheets. Reminders were sent to the patients on a weekly basis to enhance their participation. No names were required to assure confidentiality of data and data were kept confidential and utilized only for the study purpose. The study protocol was approved by the ethical research committee at the Directorate of Health Affairs in Makkah on December 7^th^, 2020 (approval number: H-02-K-076-0820-349).

The data were entered and analyzed using Statistical Package of Social Science SPSS, version 26. The descriptive statistics such as frequencies, percentages were calculated to summarize nominal and ordinal data, while mean and standard deviation were applied to describe numerical variables. Chi-squared test was performed to evaluate the association between the determinants and the outcome variables, while the means of knowledge and practice scores were compared using student t-test and one-way analysis of variance test (ANOVA) as fit. Any p-value < 0.05 was considered as an indication for a statistically significant association or difference.

## Results

Four hundred and nine (409) patients were included in this study. Their sociodemographic characteristics are presented in Table 1. Participants´ age ranged between 18 and 75 years with a mean of 42.6 and standard deviation (SD) of 13.9 years. Females represented 51.1% of the participants. The majority of the respondents were married (70.8%), Saudi individuals (94.6%) and living in urban areas (85.3%). Almost two-thirds (62.1%) were unemployed and the income of 57.2% ranged between 5,000 and 15,000 Saudi Riyals/month. Type 2 diabetes was prevalent among 85.6% of the participants. Regarding the main source of information about diabetic foot among the participants, health staff ranked first (76.8%), followed by friends/relatives (13.4%).

**Table 1:** sociodemographic characteristics of the participants (n=409)

	Frequency (n)	Percentage (%)
**Gender**		
Male	200	48.9
Female	209	51.1
**Age in years**		
Range	18-75	
Mean ± SD	42.6±13.9	
**Marital status**		
Single	80	19.6
Married	290	70.8
Divorced	22	5.4
Widowed	17	4.2
**Nationality**		
Saudi	387	94.6
Non-Saudi	22	5.4
**Place of residence**		
Urban	349	85.3
Rural	60	14.7
**Education**		
Up to intermediate school level	90	22.0
High school	107	26.2
Diploma/bachelor/+	212	51.8
**Employment status**		
Employed	155	37.9
Unemployed	254	62.1
**Family income (SR/month)**		
<5000	161	39.4
50000 - 15000	234	57.2
>15000	14	3.4

**Knowledge about diabetic foot care:** most of the participants knew correctly that their feet should be washed daily (79.5%), warm water should be used to wash their feet (72.6%), and diabetes mellitus (DM) patients should not smoke because smoking causes poor circulation affecting the feet (70.7%). On the other hand, only 29.3% could recognize that DM patients should look after their feet because they may get a foot ulcer and merely 9.5% knew correctly that if they found redness/bleeding between toes, the first thing that should be done is to be seen immediately by a chiropodist/nurse/GP (Table 2). Overall, most of the participants (72.4%) had a poor level of knowledge whereas only 4.2% expressed a good level of knowledge.

**Table 2:** responses of the participants to knowledge statements regarding diabetic foot care

	Right answer	
	No.	%
DM patients should take medication regularly because they liable to get DM complication (true)	260	63.6
DM patients should look after their feet because they may not feel a minor injury to their feet (true)	151	36.9
DM patients should look after their feet because wounds and infection may not heal quickly (true)	230	56.2
DM patients should look after their feet because they may get a foot ulcer (true)	141	29.3
DM patients should not smoke because smoking causes poor circulation affecting the feet (true)	289	70.7
How often do you think you should inspect your feet? (Daily)	284	69.4
If you found redness/bleeding between your toes what is the first thing you do? (To be seen immediately by chiropodist/nurse/GP)	39	9.5
Even if you have never had a corn/ hard skin lesion, would you do if you had one (see chiropodist)	39	9.5
How often do you think your feet should be washed (daily)	325	79.5
What temperature of water do you think you should wash your feet (warm)	297	72.6
How often do you think you should inspect the inside of your footwear for objects or torn lining (one of daily/every time footwear put on)	157	38.4

DM: diabetes mellitus

Employed patients were more knowledgeable about diabetic foot care (5.8%) compared to unemployed patients (3.1%), (p=0.046). Type 2 diabetic patients were more knowledgeable about diabetic foot care as 4.6% showed a good level of knowledge compared to 1.7% among type 1 diabetic patients, (p=0.047). The highest level of good knowledge was observed among patients whose main source of information was health staff (5.1%) while none of those whose main source was either books/journals or internet/social media implied a good level of knowledge, (p=0.026) (Table 3).

**Table 3:** factors associated with knowledge of the participants about diabetic foot care

	Knowledge level about diabetic foot care			p-value
	Low (N=296) N (%)	Medium (N=96) N (%)	Good (N=17) N (%)	
**Gender**				0.551ⱡ
Male (n=200)	142 (71.0)	51 (25.5)	7 (3.5)	
Female (n=209)	154 (73.7)	45 (21.5)	10 (4.8)	
**Age in years**				0.110*
Mean ± SD	41.59±14.48	44.97±12.36	43.59±8.49	
**Marital status**				0.071ⱡ
Single (n=80)	64 (80.0)	12 (15.0)	4 (5.0)	
Married (n=290)	206 (71.0)	73 (25.2)	11 (3.8)	
Divorced (n=22)	17 (77.3)	3 (13.6)	2 (9.1)	
Widowed (n=17)	9 (52.9)	8 (47.1)	0 (0.0)	
**Nationality**				0.441ⱡ
Saudi (n=387)	280 (72.4)	92 (23.8)	15 (3.9)	
Non-Saudi (n=22)	16 (72.7)	4 (18.2)	2 (9.1)	
**Place of residence**				0.185ⱡ
Urban (n=349)	256 (73.4)	77 (22.1)	16 (4.6)	
Rural (n=60)	40 (66.7)	19 (31.7)	1 (1.7)	
**Education**				0.662ⱡ
Up to intermediate school level (n=90)	69 (67.7)	19 (21.1)	2 (2.2)	
High school (n=107)	73 (68.2)	29 (27.1)	5 (4.7)	
Diploma/bachelor/+(n=212)	154 (72.6)	48 (22.6)	10 (4.7)	
**Employment status**				0.046ⱡ
Employed (n=155)	104 (67.1)	42 (27.1)	9 (5.8)	
Unemployed (n=254)	192 (75.6)	54 (21.3)	8 (3.1)	
**Family income (SR/month)**				0.284ⱡ
<5000 (n=161)	119 (73.9)	39 (24.2)	3 (1.9)	
50000-15000 (n=234)	169 (72.2)	52 (22.2)	13 (5.6)	
>15000 (n=14)	8 (572)	5 (35.7)	1 (7.1)	
**Type of diabetes**				0.047ⱡ
Type 1 (n=59)	49 (83.0)	9 (15.3)	1 (1.7)	
Type 2 (n=350)	247 (70.5)	87 (24.9)	16 (4.6)	
**Main source of information**				0.026ⱡ
Health staff (n=314)	223 (71.0)	75 (23.9)	16 (5.1)	
Friends/relatives (n=55)	39 (70.9)	15 (27.3)	1 (1.8)	
Books/journals (n=16)	16 (100)	0 (0.0)	0 (0.0)	
Internet/social media (n=24)	18 (75.0)	6 (25.0)	0 (0.0)	

ⱡChi-square test; *ANOVA test

**Practice regarding foot care:** almost two-thirds of the patients did not add irritants to water before foot cleaning (63.1%), did not regularly walk barefoot (62.8%), did not clean nails with sharp instruments (62.3%) and did not wear elasticated hosiery (61.4%). However, only 36.9% inspect and 36.7% wash feet on a regular basis (Table 4). Overall, 63.3% of the patients had poor level of practice-related to diabetic foot care and none of them had a good level. Although the present study investigated association between numerous factors and level of practice, none of the studied factors was significantly associated with level of practice related to diabetic foot care among the participants.

**Table 4:** responses of the participants to practice-related diabetic foot care questions

Questions	Right answer	
	No.	%
Do you inspect feet regularly? (Yes)	151	36.9
Do you wash feet regularly? (Yes)	150	36.7
Do you wash feet with warmwater? (Yes)	14	37.7
Do you trim toe nails straight across? (Yes)	151	36.9
Do you measure your feet size when last you bought footwear? (Yes)	152	37.2
Do you received advice when last you bought footwear? (Yes)	157	38.4
Did you ever inspect inside of footwear? (Yes)	152	37.2
Do you regularly walk barefoot? (No)	257	62.8
Do you clean nails with sharp instrument? (No)	255	62.3
Do you add irritants to water before feet cleaning? (No)	258	63.1
Do you wear elasticated hosiery? (No)	251	61.4

## Discussion

Diabetic foot is a relatively common health problem affecting diabetic patients and may result, if neglected, in serious complications such as amputation. Having adequate knowledge and good practices regarding foot care play an important role in preventing the development of serious diabetic foot ulcers [13,15]. Therefore, this study was carried out to assess levels of knowledge and practice regarding foot care among diabetic patients in Makkah City, Kingdom of Saudi Arabia (KSA). In the current study, most of the participants (79.5%) knew correctly that their feet should be washed daily with warm water. Similar proportion of patients was aware that they should perform proper foot hygiene in an old study done in the United Kingdom (UK) [16]. In Jeddah, Al-Gaows and Al-Zahrani observed that the majority of patients (85.4%) were unaware of suitable temperature to wash their feet, and most of them (60.1%) were unaware of how often diabetic patients should inspect their feet [17].

Most of the patients in the present study knew that diabetic patients should not smoke because smoking causes poor circulation affecting the feet. Pollock *et al*. (2004) observed that half of diabetic patients were unaware that smoking can affect the circulation to the feet [16]. Only low proportions of participants in the present study could recognize that DM patients should look after their feet because they may get a foot ulcer. Moreover, not many knew that if they found redness/bleeding between toes, the first thing should be done is to be seen immediately by a chiropodist/nurse/GP and even if they have never had a corn/hard skin lesion they should be seen by a chiropodist. Similar results have been reported in the UK [16]. However, in the Eastern Province of KSA, most of diabetic patients reported seeking immediate medical advice when they discover any foot lesions [18].

In the present study, 72.4% of the patients had a poor level of knowledge regarding foot care and only 4.2% expressed a good level of knowledge. Higher knowledge level has been observed in a recent study carried out in Iran as 15.2% showed a good level of knowledge while 84.8% of patients had a poor knowledge about diabetic foot [19]. However, lower figures of deficient knowledge regarding foot care were reported in another Saudi study (26%) [17], Iran (23.3%) [20], Nigeria (30.1%) [21], Nepal (12.3%) [13], South Africa (32.4%) [14], and Malaysia (42%) [22]. In Thailand, the mean knowledge score was 8.6 ± 2.5 out of 15 [23], and in Iraq, the mean knowledge score was 6.1 ± SD 2.6, out of 11 [24]. The differences in rates of diabetic patients` knowledge regarding foot care observed in various studies could be attributed to demographic characteristics and using different tools in assessing level of knowledge, moreover, applying different training programs on diabetic foot care by the health care professionals in various settings can affect the perceived score [25].

Among all sociodemographic factors in the current study, only employment status was significantly associated with knowledge about foot care as employed patients were more knowledgeable about diabetic foot care compared to unemployed patients. In a study carried out in Iraq (2018) [24], obese patients, smokers, patients with poor glycemic control, those living in urban area, and those belonging to high socioeconomic status were more likely to have higher level of knowledge about diabetic foot care. In other studies, carried out in Saudi Arabia [18], Tanzania [26], and India [27], duration of diabetes and patients´ educational level were significant predictors for diabetic foot knowledge level. In this study, type 2 diabetic patients were more knowledgeable about diabetic foot care compared to type 1 diabetic patients. No difference between both types was reported in another recent Saudi study carried out in Jeddah [17]. Participants whose main source of information about diabetic foot care was health staff expressed the highest level of knowledge while those whose main source of information was either books/journals or internet/social media had the lowest level of knowledge about foot care. This finding highlights the important role of healthcare staff in improving the knowledge of diabetic patients regarding foot care.

In the current study, almost two-thirds of the patients did not add irritants to water before foot cleaning, did not regularly walk barefoot, did not clean nails with sharp instruments and did not wear elasticated hosiery. However, only 36.9% and 36.7% inspect and wash feet regularly. Similar results in most practices have been obtained in another Saudi study carried out in Dammam, however, better practice regarding inspection and washing feet with warm water regularly have been observed [18]. Surprisingly, finding that only one-third of patients reported washing of feet regularly is lower than expected as people in Saudi Arabia are Muslims and they have to wash their feet five times daily for praying. Possibly, they considered washing feet as a separate act from washing for praying.

Overall, in the current study, 63.3% of patients had a poor level of practice related to diabetic foot care and none had a good level. Similar to these findings, in Malaysia, 61.8% of diabetic patients had a poor diabetic foot care practice [22] and in Thailand [23], 60% of the patients expressed poor diabetic foot care practices. However, in Iran [28], 50.4% of patients expressed a good level of practice regarding diabetic care. Perhaps, using different tools and cut-off values in the previously mentioned study and our study justify the difference in the findings. In agreement with others in Iran [28], Iraq [24], Sri Lanka [29], Bangladesh [30], Tanzania [26], and South Africa [14], there was a significant association between level of knowledge of foot care and foot care practices in the current study.

In the present study, none of the investigated factors was associated with level of daily practice towards foot care. In a recent Iranian study [19], residence place, marital status, and history of hospital admission due to diabetic foot were determinants of foot care good practice. In Thailand [23], gender, family history of diabetes, socioeconomic and marital status were significantly associated with level of diabetic foot care practice among patients. The present study encountered three main potential limitations. First, patients were recruited from a single center in Makkah, which could affect the generalizability of results over the entire diabetic population in Makkah. Second, the cross-sectional design of the study cannot determine the direction of causal relationships. Finally, some important elements were not investigated in this study; mainly glycemic control and family support.

## Conclusion

Low level of knowledge and poor practice of foot care are common among diabetic patients attending outpatient clinics of Heraa Diabetic Center, Ministry of Health in Makkah, KSA. Employed patients, type 2 diabetes patients and those whose main source of information was health staff were more likely to express good foot care knowledge than their counterparts. There is a need for educational intervention addressing the importance of diabetic foot self-care practices among diabetic population.

### What is known about this topic

- In Saudi Arabia, 55.2% of the diabetic patients developed complications related to diabetic foot;
- Awareness about proper foot care can reduce complications, admission, and amputation rates.

### What this study adds

- Low level of knowledge and poor practice of foot care are common among diabetic patients in Saudi Arabia;
- Health staff plays a significant role in raising patients´ awareness regarding foot self-care.

## References

[1] Naeem Z (2015). Burden of diabetes mellitus in Saudi Arabia. Int J Health Sci (Qassim).

[2] World Health Organization (1994). Prevention of diabetes mellitus: report of a WHO study group [meeting held in Geneva from 16 to 20 November 1992].

[3] Al Dawish MA, Robert AA, Braham R, Al Hayek AA, Al Saeed A, Ahmed RA (2016). Diabetes mellitus in Saudi Arabia: a review of the recent literature. Curr Diabetes Rev.

[4] Ahmad S (2013). Diabetes: an old disease, a new insight.

[5] Alanazi FK, Alotaibi JS, Paliadelis P, Alqarawi N, Alsharari A, Albagawi B (2018). Knowledge and awareness of diabetes mellitus and its risk factors in Saudi Arabia. Saudi Med J.

[6] Funnell M, Anderson RM (2004). Empowerment and self-management of diabetes. Clinical Diabetes.

[7] Ahola AJ, Groop P-H (2013). Barriers to self-management of diabetes. Diabet Med.

[8] Gillies CL, Abrams KR, Lambert PC, Cooper NJ, Sutton AJ, Hsu RT (2007). Pharmacological and lifestyle interventions to prevent or delay type 2 diabetes in people with impaired glucose tolerance: systematic review and meta-analysis. BMJ.

[9] Reiber GE, King H, World Health Organization (1991). Guidelines for the development of a national programme for diabetes mellitus.

[10] Peyrot M, Rubin RR, Lauritzen T, Snoek FJ, Matthews DR, Skovlund SE (2005). Psychosocial problems and barriers to improved diabetes management: results of the cross-national Diabetes Attitudes, Wishes and Needs (DAWN) Study. Diabet Med.

[11] Steed L, Cooke D, Newman S (2003). A systematic review of psychosocial outcomes following education, self-management and psychological interventions in diabetes mellitus. Patient Educ Couns.

[12] Al-Jarallah M, Alqahtani M, Alshahrani S, Alshahrani N, Mohammed Alshehri A (2020). Knowledge and practice of diabetic foot care among diabetic patients in Aseer region, Saudi Arabia. IJMDC.

[13] Gautam A, Bhatta DN, Aryal UR (2015). Diabetes related health knowledge, attitude and practice among diabetic patients in Nepal. BMC Endocr Disord.

[14] Ralineba T, Netshikweta ML, Shilubane NH (2015). Knowledge and practices associated with diabetes among patients with chronic diabetes mellitus in rural areas of Vhembe District, Limpopo Province, South Africa. Journal of Human Ecology.

[15] Meaney B (2012). Diabetic foot care: prevention is better than cure. J Ren Care.

[16] Pollock RD, Unwin NC, Connolly V (2004). Knowledge and practice of foot care in people with diabetes. Diabetes Res Clin Pract.

[17] Gaows F, Alzahrani A (2019). Knowledge and practice of foot care among diabetic patients attending diabetic care center in Jeddah City. Int J Med Rev Case Rep.

[18] Al-Hariri MT, Al-Enazi AS, Alshammari DM, Bahamdan AS, AL-Khtani SM, Al-Abdulwahab AA (2017). Descriptive study on the knowledge, attitudes and practices regarding the diabetic foot. J Taibah Univ Med Sci.

[19] Pourkazemi A, Ghanbari A, Khojamli M, Balo H, Hemmati H, Jafaryparvar Z (2020). Diabetic foot care: knowledge and practice. BMC Endocr Disord.

[20] Kafaie P, Noorbala MT, Soheilikhah S, Rashidi M (2012). Evaluation of patients´ education on foot self-care status in diabetic patients. Iran Red Crescent Med J.

[21] Desalu OO, Salawu FK, Jimoh AK, Adekoya AO, Busari OA, Olokoba AB (2011). Diabetic foot care: self reported knowledge and practice among patients attending three tertiary hospital in Nigeria. Ghana Med J.

[22] Muhammad-Lutfi AR, Zaraihah MR, Anuar-Ramdhan IM (2014). Knowledge and practice of diabetic foot care in an in-patient setting at a tertiary medical center. Malays Orthop J.

[23] Kim SY, Hongsranagon P (2008). Preventive behaviors regarding foot ulcers in diabetes type II patients at BMA Health Center no 48, Bangkok, Thailand. Journal of Health Research.

[24] Saber HJ, Daoud AS (2018). Knowledge and practice about the foot care and the prevalence of the neuropathy among a sample of type 2 diabetic patients in Erbil, Iraq. J Family Med Prim Care.

[25] Hamidah H, Santhna LP, Ruth Packiavathy RD, Suraya AM, Yap WC, Samsiah M (2012). Foot care strategy for the newly diagnosed DM type 2 patients with low educational and socio-economic background: a step towards future. Clin Ter.

[26] Chiwanga FS, Njelekela MA (2015). Diabetic foot: prevalence, knowledge, and foot self-care practices among diabetic patients in Dar es Salaam, Tanzania-a cross-sectional study. J Foot Ankle Res.

[27] George H, Rakesh P, Krishna M, Alex R, Abraham VJ, George K (2013). Foot care knowledge and practices and the prevalence of peripheral neuropathy among people with diabetes attending a secondary care rural hospital in Southern India. J Family Med Prim Care.

[28] Khamseh ME, Vatankhah N, Baradaran HR (2007). Knowledge and practice of foot care in Iranian people with type 2 diabetes. Int Wound J.

[29] Perera DP, De Silva RE, Perera WLSP (2013). Knowledge of diabetes among type 2 diabetes patients attending a primary health care clinic in Sri Lanka. East Mediterr Health J.

[30] Saleh F, Mumu SJ, Ara F, Begum HA, Ali L (2012). Knowledge and self-care practices regarding diabetes among newly diagnosed type 2 diabetics in Bangladesh: a cross-sectional study. BMC Public Health.

